# Factors Contributing to Delayed Initiation of Appropriate Antimicrobial Treatment in Patients With Streptococcal Bacteremia

**DOI:** 10.1093/cid/ciae317

**Published:** 2024-06-06

**Authors:** Nicolas Fourré, Benoit Guery, Matthaios Papadimitriou-Olivgeris

**Affiliations:** Infectious Diseases Service, Lausanne University Hospital, Lausanne, Switzerland; Infectious Diseases Service, Lausanne University Hospital, Lausanne, Switzerland; Infectious Diseases Service, Lausanne University Hospital, Lausanne, Switzerland; Infectious Diseases Service, Cantonal Hospital of Sion and Institut Central des Hôpitaux, Sion, Switzerland

To the Editor—In their insightful commentary, Dequidt et al [1] raise important questions regarding the impact of appropriate antimicrobial treatment on the survival of patients with streptococcal bacteremia [2].

Dequidt et al [1] are correct that the cited article [3] showed a numerical but not statistically significant impact of appropriate antimicrobial treatment on mortality among patients with streptococcal bacteremia. The positive impact of appropriate antimicrobial treatment on survival was shown in a meta-analysis on invasive pneumococcal disease, where inappropriate empiric therapy was associated with increased mortality (*P* = .002; 95% confidence interval [CI]: 1.27–3.00) [4]. This meta-analysis demonstrated that, although appropriate antimicrobial treatment is an important predictor of survival, only 8 out of 190 studies included this parameter in their analysis. Furthermore, among patients presenting with suspected infection, only a minority will have bacteremia [5], of which a small percentage will be due to streptococci [6, 7]. Thus, only observational studies, with their inherent biases, could evaluate, in a posteriori manner, the impact of appropriate antimicrobial treatment on mortality among patients with streptococcal bacteremia.

In our study, 820 (95%) episodes received appropriate antimicrobial treatment within 48 hours of the first positive blood culture, which is comparable to the literature [2, 7]. There may be several reasons for the remaining 41 (5%) episodes not receiving appropriate antimicrobial treatment within the same timeframe.

The delay in communication to the treating physician of the positive blood culture result beyond the 48-hour mark could be the reason for a delayed initiation of antimicrobial treatment in a proportion of patients. Although the time from blood culture collection and the moment of communication of the result could not be calculated, we found that in 81 (9%) of episodes, the time to blood culture positivity exceeded 24 hours. A time to positivity of more than 24 hours was more common in episodes that did not receive appropriate antimicrobial treatment compared to those that did (24% vs 9%; *P* = .005) (Table 1). If we consider the duration from blood culture collection to insertion in the BacT/ALERT System (bioMerieux, Marcy l'Etoile, France), the time required for species identification, and the absence of species identification from 19:00 to 07:00, we could infer that for some patients, the treating physician was informed of the presence of positive blood culture after the 48-hour mark [8].

**Table 1.** Comparison of Episodes With and Without Appropriate Antimicrobial Treatment Within 48 h

	No Appropriate Antimicrobial Treatment (n = 41)		Appropriate Antimicrobial Treatment (n = 820)		*P*
Demographics					
Male sex	27	66%	559	68%	.734
Age (y)	74	63–85	66	51–76	.017
Age >60 y	31	75%	506	62%	.097
Charlson Comorbidity Index	3	2–4	2	1–5	.158
Charlson Comorbidity Index >4	23	56%	411	50%	.523
Beta-lactam allergy	12	29%	69	8%	<.001
Microbiological data					
Polymicrobial bloodstream infection	10	24%	219	27%	.857
Increased exposure or resistant to penicillin	10	24%	76	9%	.005
Penicillin resistance	7	17%	30	4%	.001
Time to positivity >24 h	10	24%	71	9%	.003
Follow-up blood cultures	22	54%	625	76%	.002
Persistent bacteremia (≥48 h)	2	9%	2	.3%	.006
Infection data					
Fever	28	68%%	718	88%	.001
Sepsis	15	37%	282	34%	.866
Septic shock	4	10%	116	14%	.642
Management					
Infectious diseases consultation within 48 h	20	49%	588	72%	.003
Source control					
Not warranted	22	54%	519	63%	
Warranted and performed within 48 h	11	27%	188	23%	
Warranted but not performed within 48 h	8	20%	113	14%	.418
Death within 14 d	11	27%	54	7%	<.001

Data are depicted as number and percentage or median and Q1–3.

Antimicrobial resistance is a common reason for inappropriate antimicrobial treatment. However, streptococci are rarely resistant to empiric beta-lactam treatment [4, 9]. In this study, penicillin resistance among isolated streptococci was found in 4% of cases, with a higher incidence among episodes that did not receive appropriate antimicrobial treatment compared to those that did (17% vs 4%; *P* < .001).

Among the 11 patients who died within 14 days without receiving appropriate antimicrobial treatment within 48 hours, 3 passed away within the initial 48-hour window. Another 2 patients initially received antimicrobial treatment to which the isolated *Streptococcus* spp. was resistant. For the remaining 6 patients, antimicrobial treatment commenced after the initial 48 hours, immediately following the receipt of blood culture results. Apart from the initial 3 patients, 4 more passed away between 48 and 96 hours, whereas the remaining 4 succumbed after 96 hours.

Another aspect highlighted by Dequidt et al concerns the absence of positive blood cultures at the 48-hour mark following the initial positive blood culture, despite the absence of appropriate antimicrobial treatment [1]. There are several explanation for this discrepancy: first, not all episodes had follow-up blood cultures. Second, apart from endocarditis and endovascular infections in general, for other foci of infection bacteremia is not continuous [10]. Third, some patients who received appropriate antimicrobial treatment after 48 hours could have had follow-up blood cultures drawn subsequent to the initiation of that treatment, explaining the clearance of bacteremia. In our study, a higher rate of positive follow-up blood cultures for at least 48 hours was observed among episodes without appropriate antimicrobial treatment compared to those with it (9% vs 0.3%; *P* < .001) [2].

Finally, we concur that results from observational studies should be interpreted with caution, particularly regarding parameters reflecting medical decisions, such as antimicrobial treatment or source control interventions, as these decisions are based on complex evaluations not fully captured during data collection. However, the effect of appropriate antimicrobial treatment on the mortality of various infections and pathogens has consistently been shown to be one of the most important interventions for improving outcomes [11]. Nonetheless, as noted by Dequidt et al [1], in patients with suspected infection and in the absence of an evident site of infection, sepsis, and neutropenia, antimicrobial treatment can be safely withheld, as initiating antimicrobial treatment in all such patients could lead to unnecessary consequences at both the individual and society levels, including gut microbiota dysregulation, antimicrobial-associated side effects, and increase in antimicrobial resistance.
